# Jungian personality type preferences of female and male Hungarian leaders

**DOI:** 10.3389/fpsyg.2023.1222568

**Published:** 2023-10-05

**Authors:** Edit Szathmári, Andrea Czibor, Richard Bents, Zsolt Péter Szabó, Orhidea Edith Kiss

**Affiliations:** ^1^Doctoral School of Psychology, ELTE Eötvös Loránd University, Budapest, Hungary; ^2^Institute of Psychology, ELTE Eötvös Loránd University, Budapest, Hungary; ^3^Institute of Psychology, University of Pécs, Pécs, Hungary; ^4^Future Systems Consulting Inc., St. Paul, MN, United States; ^5^Department of Ergonomics and Psychology, Faculty of Economic and Social Sciences, Budapest University of Technology and Economics, Budapest, Hungary

**Keywords:** leadership, executive leaders, female leaders, Jungian personality type preferences, Golden Profiler of Personality

## Abstract

In this study, we investigated the personality type preferences of female and male Hungarian non-managerial individual contributors, middle managers, and executives. We aimed to investigate the preferences among successful females and males (i.e., executives) compared to non-executives. The preference distinctions between successful females and males were also analyzed. We conducted a cross-sectional analysis using the Jungian-based Golden Profiler of Personality (GPOP) questionnaire (*N* = 5,376; 2,678 females, 2,698 males; average age 35.98 with an *SD* = 8.977). Executives scored higher in extraversion, intuition, thinking, perceiving, and calm preferences compared to middle managers and individual contributors while scoring lower in sensing and tense preferences. Extraversion, intuition, and feeling preferences were more prevalent among female executives than both male executives and women in general. Our findings suggest that Hungarian female executives' personality preferences align with either stereotypically feminine traits (intuitive and feeling) or male executive-like preferences (extraverted, sensing, thinking, and judging combination). We also discussed the influence of cultural norms and expectations on the personality preferences of female and male executives. Our results are in line with prior research conducted in the Western context, however, the gender differences are more striking. We concluded that men have a reasonable chance of success across a spectrum of personality preferences as they ascend the hierarchy, while women need to exhibit specific preferences to be successful on the same journey. The self-descriptive and cross-sectional nature of our data spell limitations, therefore we suggest conducting future longitudinal studies, including explanatory and contingency variables (e.g. perceived cultural norms).

## 1. Introduction

Hungary's State Audit Office recently released a report titled “Signs of Pink Education in Hungary” (Aradi, 2022). The report suggests that the overrepresentation of women in universities could potentially result in demographic challenges, as highly educated women might encounter difficulties in finding equally educated partners. While the report expresses concerns about potential disadvantages for men due to “pink education,” the reality is that gender inequality and the underrepresentation of women in positions of power remain persistent issues in Hungary (Nagy, 2012). The main aim of our study was to explore gender-related differences in the personality preferences of leaders. Do they conform to stereotypical aspects of the female leadership style [referred to as the “female leadership advantage,” as discussed by Offermann and Foley (2020)] to achieve success, or do they copy male leadership styles?

### 1.1. Personality traits and personality preferences

A longstanding question in leadership literature revolves around whether personality preferences are linked to leadership emergence and effectiveness (Furnham and Crump, 2015). Personality preferences are the person's preferences for using his/her own perceptions and judgments in all daily activities (Gentry et al., 2007). This concept originates from the personality theory of Jung. Jung (1971) defined four psychological functions -thinking, feeling, sensing, and intuition- and described two basic types, the extrovert, and the introvert, based on these functions. While Jung portrayed the differences in preferences as types, contemporary tools such as the Myers-Briggs Type Indicator (MBTI; Briggs and Myers, 1976) and the Golden Profiler of Personality (GPOP; Golden, 2010) treat preferences as continuous scales.

Jung's concept, expanded by Briggs and Myers (1976) and later by Golden (2010), encompasses four pairs of preferences: (1) Source of energy: This addresses where an individual derives their energy from. Extraversion (E) means that the person gains energy from the outer world, relationships, and events, whereas Introversion (I) means that the person gains energy from the inner world, thoughts, and feelings. (2) Perception: This concerns the approach to collecting data and forming information based on it. Sensing (S) involves gathering concrete and observable facts through sensory organs, while intuition (N) involves focusing on meanings, relationships, and patterns beyond directly observable facts. (3) Decision-making: This pertains to the foundation of conclusions and the manner of decision-making. Thinking (T) signifies decisions based on objective logic and an objective process while Feeling (F) indicates decisions grounded in subjective logic, relationships, and interpersonal considerations. (4) Lifestyle: This answers how individuals engage with the external world and encompasses their overall way of living. Perception (P) characterizes a flexible, adaptable lifestyle that prefers data and experiences collected via sensing or intuition. Judgement (J) is marked by an organized, planned lifestyle using either. Golden (2010) extended this framework by introducing a fifth element. (5) Stress preference: This addresses how individuals approach and respond to general life stressors. A preference for Tense (Te) indicates heightened concern and insecurity in the face of stressors, while a Calm (C) preference reflects a more optimistic and confident approach to stress.

### 1.2. The associations between personality preferences and leadership

The main conclusion drawn from studies utilizing the Jungian personality model (Myers and McCaulley, 1985) in this domain is that while all Jungian types are present among managers, Thinking and Judging types exhibit a notably high representation. This prevalence of TJ appears consistent across different organizational levels. However, E-I and S-N preferences do not exhibit a distinct pattern among top-level or middle-level managers, nor among managers overall (e.g., Mosley and Pietri, 1985; Brightman and Sayeed, 1990; Campbell and Kain, 1990; Cabral and Joyce, 1991; Johnson, 1992).

Research from the 1990s highlighted a distinction: among middle-level leaders (managers), E, S, T, and J preferences were more prevalent, while among higher-level leaders (executives), E, N, T, and J preferences were overrepresented (Carland and Carland, 1992; Reynierse, 1993, 1997). Recent studies further substantiated this, confirming that managers occupying elevated positions within the corporate hierarchy exhibit higher scores in Extraversion, Intuition, and Thinking when compared to lower-level managers (Moutafi et al., 2007). In a study by Furnham and Crump (2015) encompassing three distinct managerial levels (individual contributors, middle-level managers, and executives), the findings indicated that Thinking scores for middle managers and executives were significantly higher than those of non-managers, portraying the most robust distinction between leaders and non-leaders. Additionally, the study revealed that executives displayed markedly higher Perceiving scores compared to non-managers and middle managers (cf. Gardner and Martinko, 1996). Sensing scores were the highest among non-managers and the lowest among middle managers, while executives were midway.

Given these diverse findings in the literature, a thorough exploration of preference disparities across managerial levels warrants further investigation.

### 1.3. Gender effects on leadership behavior and preferences

The impact of gender on leadership is a wide-ranging and dynamic field of research (Eagly and Karau, 1991; Paustian-Underdahl et al., 2014; Badura et al., 2018). Within this domain, numerous studies tackle the paradox between the persistently low representation of female leaders and their perceived effectiveness, employing various leadership style theories (Samuelson et al., 2019; Shen and Joseph, 2021). According to Cheung and Halpern (2010) while leadership roles promote similarities in male and female leaders, women typically exhibit a more democratic, participative, and collaborative leadership style. Their review of recent studies shows that women often embrace a relational leadership approach marked by mutuality and equality (Chrobot-Mason et al., 2014). These tendencies can be linked to Jungian Thinking and Feeling preferences, wherein notable gender disparities are observable: men tend to score significantly higher on the Thinking scale, whereas women on the Feeling scale (e.g., Vacha-Haase and Thompson, 2002; Johnson et al., 2009).

Regarding the Perception global scale, Hayes et al. (2004) investigated the evidence behind the common belief that women are more intuitive than men. They have found that contrary to the stereotype, there was no difference between male and female managers in Intuition. Moreover, among non-managers men were more Intuitive than women.

Turning to stress, Nguyen et al. (2012) observed that women had a significantly higher level of stress perception. Similarly, Tomiak et al. (1997) found that the managerial career trajectory presents a more stressful journey for women compared to men.

When examining gender effects within the context of leadership and personality preferences, there are diverse and sporadic results. Some studies discovered no gender-based disparities in the personality preferences of male and female leaders (e.g., Hansson and Andersen, 2007; Kummerow and Herk, 2011; Cohen et al., 2013; Gilal et al., 2016), whereas others did (e.g., Brandt and Laiho, 2013).

The majority of these investigations explored gender-related disparities in personality preferences within North American and Western European samples. In contrast, our study was conducted in Hungary, a cultural context that markedly differs from those contexts.

### 1.4. The Hungarian context

In Hungary, gender inequalities persist, encompassing the underrepresentation of women in management and other political and economic leadership positions (Nagy, 2012). This issue has remained persistent since the democratic system changes in 1989 (Nagy, 2012; Nagy et al., 2022). The European Institute for Gender Equality highlights that Hungary's Gender Equality Index consistently falls below the European Union's average (European Institute for Gender Equality, 2022).

Prominent factors contributing to this situation are historical, traditional, and cultural (Deloitte Slovenia, 2014). Drawing from Hofstede's and GLOBE survey outcomes, Nagy and Vicsek (2014) propose that Hungary exhibits a masculine culture, wherein stereotypically masculine attitudes and behaviors hold greater value than feminine ones within the realm of work. Conservative gender role attitudes are dominant, and there is a perception of incompatibility between child-rearing and pursuing a career (Scharle, 2015). These factors contribute to the prevalent bias against female leaders, characterizing them as less competent in comparison to their male counterparts (Nagy and Vicsek, 2008). Furthermore, female leaders are disproportionately represented in areas where organizations are feminized, implying that their organizational authority primarily extends over female cohorts. This arrangement perpetuates the status quo, thereby minimizing the influence of female leaders (Nagy, 2012).

Within this context, what personality preferences must successful women exhibit? Are they compelled to conform to the stereotypes associated with female leadership? Alternatively, should they demonstrate traditional male traits often linked to leadership? Or, as a third option, do they have “to be ‘feminine' and hard at the same time,” as articulated by Nagy (2012, p. 240), creating a double bind for female leaders?

### 1.5. Aims of the present study

In the present study, we utilize cross-sectional data from Hungarian female and male employees across various hierarchy levels to reveal personality preference disparities between female and male non-managerial individual contributors, middle managers, and executives. Specifically, we seek to address the following research questions: (1) Do personality preferences among successful females and males (i.e., those in executive positions) differ from those of female and male middle managers and non-managerial employees? (2) Are the personality preferences of successful females distinct from those of successful males?

Based on the literature reviewed above, we have formulated the following hypothesis and research question:

H1. Jungian personality preferences differ by hierarchy levels: higher levels are associated with higher levels of Extraversion, Thinking, Judgment, and Calm scores, and lower levels of Tense.

RQ1. Are the personality preferences and personality types of female and male leaders different? Do successful females have to fit into the female leader stereotype, show a male leadership style, or a mixture of the two (feminine and hard at the same time)?

## 2. Materials and method

### 2.1. Participants and procedure

Participants were asked to complete the Golden Profiler of Personality (GPOP) questionnaire aimed at assessing personality preferences. To the questionnaire, 5376 responses were recorded by 5282 different participants. The sample consisted of 50.2% male and 49.8% female participants, spanning ages from 18 to 88 years, with an average age of 36 years (*SD* = 8.98). About half of the respondents (52%, 2781 responses) were individual contributors (i.e., non-managers), comprising 39.4% male and 60.5% female participants. Additionally, 37% (1968 responses) held middle management roles with 60% male and 40% female representation, and 12% (627 responses) occupied executive positions (comprising 69.7% male and 30.3% female participants).

The data was collected by an external consultancy company within a developmental context spanning the years 2006 to 2018. A wide variety of industries and companies were represented in the sample, including for instance telecommunication (n = 501), energy sector (n = 400), and educational companies (n = 167). Participants received detailed information regarding the GPOP and were required to provide their informed consent before participating. Feedback on participants' results was provided during the development programs. Notably, this dataset was also utilized for The Hungarian validation of the GPOP (Czibor et al., 2019).

### 2.2. Measures

#### 2.2.1. Jungian personality preferences

Jungian personality preferences were assessed using an online version of the Golden Profiler of Personality (GPOP; Golden, 2010) questionnaire. The GPOP is a 126-item self-reported personality survey combining Jung's theory of psychological type and the Big Five model of personality. The GPOP measures five global scales and ten subscales (Source of energy: Extraversion vs. Introversion; Perception: Sensing vs. Intuition; Decision making: Thinking vs. Feeling; Lifestyle: Perception vs. Judgement; Stress: Tense vs. Calm). The factor structure, reliability, and validity of the Hungarian adaptation were tested. Confirmatory factor analyses confirmed the factor structure of the Hungarian version. The global scales and the subscales showed high internal consistency, and the correlations between the GPOP scales and broad personality traits were consistent with the Jungian theory and with earlier research findings (see Czibor et al., 2019). In summary, the Hungarian version of the GPOP is a reliable and valid tool for measuring personality preferences.

#### 2.2.2. Demographic variables

Participants provided the following demographic information: company, gender, and age. Gender was used as the main independent variable in our study, and age was used as a control variable. Information about the participants' companies was not used in the analysis.

### 2.3. Data-analytical approach

The statistical data analysis was carried out with IBM SPSS Statistics for Windows, Version 25.0 (https://scicrunch.org/resolver/RRID:SCR_019096). To address our hypothesis and research question, two-way ANOVAs were conducted to compare the main effects of gender (male vs. female) and hierarchical level (individual contributor vs. middle manager vs. executive) and the interaction effect between gender and hierarchical level on the subscales of the GPOP (Extraversion, Introversion, Sensing, Intuition, Thinking, Feeling, Perception, Judgement, Tense, Calm). The significance level was adjusted to 0.0017 (i.e., dividing the 0.05 threshold by thirty) with Bonferroni correction to account for multiple comparisons. Age was included as a control variable.

To further investigate our research question, we conducted a cross-tabulation analysis using a chi-square test to determine the associations between categorical variables, i.e., gender, hierarchical levels, and personality profiles (ENFJ, ENFP, ENTJ, ENTP, ESFJ, ESFP, ESTJ, ESTP, INFJ, INFP, INTJ, INTP, ISFJ, ISFP, ISTJ, ISTP).

## 3. Results

Descriptive statistics of preferences for the groups (male and female individual contributors, middle managers, and executives) are shown in Table 1.

**Table 1:** Means and standard deviation of the study variables.

**Variables**	**Groups**	**Non-managers *M* (*SD*)**	**Middle managers *M* (*SD*)**	**Executives *M* (*SD*)**	**Total**
DV 1: Extraversion	Male	0.37 (0.21)	0.40 (0.21)	0.44 (0.21)	0.39 (0.21)
	Female	0.40 (0.21)	0.44 (0.21)	0.49 (0.20)	0.42 (0.21)
	Total	0.39 (0.21)	0.41 (0.21)	0.45 (0.21)	0.40 0.21)
DV 2: Introversion	Male	0.19 (0.15)	0.17 (0.13)	0.17 (0.12)	0.18 (0.14)
	Female	0.21 (0.16)	0.18 (0.14)	0.17 (0.13)	0.20 (0.15)
	Total	0.20 (0.15)	0.17 (0.13)	0.17 (0.12)	0.19 (0.14)
DV 3: Sensing	Male	0.31 (0.17)	0.32 (0.15)	0.29 (0.16)	0.31 (0.16)
	Female	0.31 (0.17)	0.32 (0.17)	0.27 (0.17)	0.31 (0.17)
	Total	0.31 (0.17)	0.32 (0.16)	0.28 (0.16)	0.31 (0.16)
DV 4: Intuition	Male	0.24 (0.15)	0.22 (0.14)	0.28 (0.17)	0.24 (0.15)
	Female	0.25 (0.17)	0.25 (0.16)	0.32 (0.18)	0.26 (0.17)
	Total	0.24 (0.16)	0.24 (0.15)	0.29 (0.18)	0.25 (0.16)
DV 5: Thinking	Male	0.30 (0.15)	0.31 (0.14)	0.32 (0.15)	0.31 (0.14)
	Female	0.19 (0.12)	0.21 (0.13)	0.22 (0.12)	0.20 (0.12)
	Total	0.23 (0.14)	0.27 (0.14)	0.29 (0.15)	0.25 (0.15)
DV 6: Feeling	Male	0.24 (0.14)	0.23 (0.12)	0.24 (0.13)	0.24 (0.13)
	Female	0.40 (0.17)	0.37 (0.16)	0.37 (0.17)	0.39 (0.17)
	Total	0.34 (0.17)	0.29 (0.16)	0.28 (0.15)	0.31 (0.17)
DV 7: Judging	Male	0.41 (0.17)	0.42 (0.17)	0.41 (0.18)	0.41 (0.17)
	Female	0.41 (0.18)	0.43 (0.18)	0.40 (0.18)	0.41 (0.18)
	Total	0.41 (0.17)	0.42 (0.17)	0.40 (0.18)	0.41 (0.17)
DV 8: Perceiving	Male	0.18 (0.12)	0.16 (0.12)	0.20 (0.14)	0.18 (0.13)
	Female	0.20 (0.13)	0.20 (0.14)	0.22 (0.13)	0.20 (0.14)
	Total	0.19 (0.13)	0.18 (0.13)	0.20 (0.14)	0.19 (0.13)
DV 9: Tense	Male	0.17 (0.14)	0.12 (0.10)	0.11 (0.10)	0.14 (0.12)
	Female	0.22 (0.15)	0.16 (0.12)	0.14 (0.12)	0.19 (0.15)
	Total	0.20 (0.15)	0.14 (0.11)	0.12 (0.11)	0.17 (0.14)
DV 10: Calm	Male	0.41 (0.20)	0.46 (0.18)	0.50 (0.18)	0.44 (0.19)
	Female	0.37 (0.18)	0.45 (0.18)	0.49 (0.18)	0.40 (0.19)
	Total	0.39 (0.19)	0.45 (0.18)	0.49 (0.018)	0.42 (0.19)

Table 2 shows the results of the two-way ANOVAs.

**Table 2:** Personality preferences as functions of gender and hierarchy level.

	***F***	***p***	** $\eta_{p}^{2}$ **
**DV: Extraversion**			
Gender	33.44	<0.001	0.006
Hierarchy level	47.71	<0.001	0.017
Gender X hierarchy level	1.16	0.31	0.000
**DV: Introversion**			
Gender	2.46	0.12	0.000
Hierarchy level	36.58	<0.001	0.013
Gender X hierarchy level	0.81	0.45	0.000
**DV: Sensing**			
Gender	1.21	0.27	0.000
Hierarchy level	16.27	<0.001	0.006
Gender X hierarchy level	0.69	0.50	0.000
**DV: Intuition**			
Gender	25.69	<0.001	0.005
Hierarchy level	35.09	<0.001	0.013
Gender X hierarchy level	2.71	0.07	0.001
**DV: Thinking**			
Gender	512.59	<0.001	0.087
Hierarchy level	22.42	<0.001	0.008
Gender X hierarchy level	4.72	0.009	0.002
**DV: Feeling**			
Gender	769.80	<0.001	0.125
Hierarchy level	23.26	<0.001	0.009
Gender X hierarchy level	2.59	0.08	0.001
**DV: Judging**			
Gender	0.00	0.10	0.000
Hierarchy level	5.05	0.006	0.002
Gender X hierarchy level	0.30	0.74	0.000
**DV: Perceiving**			
Gender	34.10	<0.001	0.006
Hierarchy level	11.71	<0.001	0.004
Gender X hierarchy level	0.89	0.41	0.000
**DV: Tense**			
Gender	62.31	<0.001	0.011
Hierarchy level	105.56	<0.001	0.038
Gender X hierarchy level	3.68	0.03	0.001
**DV: Calm**			
Gender	9.02	0.003	0.002
Hierarchy level	111.07	<0.001	0.040
Gender X hierarchy level	3.31	0.04	0.001

Age was controlled for in the analysis.

The main effect of gender was significant for the following personality preferences: extraversion, intuition, thinking, feeling, perceiving, tense, and calm. Female participants scored higher on extraversion, intuition, feeling, perceiving, and tense, and scored lower on thinking and calm compared to male participants (see Table 1 for descriptive statistics).

The main effect of hierarchy level was significant for all personality preferences but Judgement (the analysis for this variable failed to reach the Bonferroni-corrected significance level of 0.0017). Pairwise comparisons (Bonferroni test) show that executives scored significantly higher on extraversion, thinking, calm, intuition, and perceiving, and lower on sensing and judging than middle managers and individual contributors. Middle managers scored significantly higher on extraversion, thinking, and calm compared to individual contributors. Both executives and middle managers scored lower on introversion, feelings, and tense than individual contributors, but there were no significant differences between the two managerial levels (see Table 1 for descriptive statistics). *P*s are <0.001 except for significant differences between the executives' and middle managers' thinking scores (*p* =0.03), and judging scores (*p* =0.011). The interaction terms between gender and hierarchy level did not reach significance (*p*s were between 0.03 and 0.74; Bonferroni-corrected *p* level <0.0017). These findings partially confirmed H1: personality preferences differed by hierarchy levels and the differences were mostly in the expected directions. Executives displayed higher scores on extraversion, thinking, and calm, and lower scores on tense compared to the other two hierarchical levels. Unexpectedly, their scores on Judging were lower than the two other groups.

Table 3 shows the three-way cross-tabulation of categorical variables (i.e., gender, hierarchical level, personality type). Gender (male vs. female) was used as a primary category and was then broken down by hierarchical level. Rows in Table 3 indicate the frequency of the total number of each personality profile, while cells consist of the cross-tabulation of hierarchical levels by gender.

**Table 3:** Cross-tabulation of participants' personality types by gender and hierarchy level.

	***N***	**ENFJ**	**ENFP**	**ENTJ**	**ENTP**	**ESFJ**	**ESFP**	**ESTJ**	**ESTP**	**INFJ**	**INFP**	**INTJ**	**INTP**	**ISFJ**	**ISFP**	**ISTJ**	**ISTP**
**Overall**	5,376	674 (12.5%)	475 (8.8%)	419 (7.8%)	180 (3.3%)	1,063 (19.8%)	140 (2.6%)	919 (17.1%)	86 (1.6%)	140 (2.6%)	103 (1.9%)	102 (1.9%)	74 (1.4%)	451 (8.4%)	60 (1.1%)	446 (8.3%)	44 (0.8%)
**Males**	2,698	218 (8.1%)	161 (6.0%)	311 (11.5%)	128 (4.7%)	357 (13.2%)	41 (1.5%)	687 (25.5%)	62 (2.3%)	48 (1.8%)	41 (1.5%)	71 (2.6%)	53 (2.0%)	139 (5.2%)	19 (0.7%)	329 (12.2%)	33 (1.2%)
IC	1,098	91 (8.3%)	61 (5.6%)	114 (10.4%)	49 (4.5%)	144 (13.1%)	20 (1.8%)	246 (22.4%)	31 (2.8%)	25 (2.3%)	22 (2.0%)	37 (3.4%)	28 (2.6%)	73 (6.6%)	5 (0.5%)	137 (12.5%)	15 (1.4%)
MM	1,163	93 (8.0%)	60 (5.2%)	130 (11.2%)	50 (4.3%)	163 (14.0%)	15 (1.3%)	330 (28.4%)	24 (2.1%)	19 (1.6%)	12 (1.0%)	21 (1.8%)	17 (1.5%)	55 (4.7%)	9 (0.8%)	152 (13.1%)	13 (1.1%)
EX	437	34 (7.8%)	40 (9.2%)	67 (15.3%)	29 (6.6%)	50 (11.4%)	6 (1.4%)	111 (25.4%)	7 (1.6%)	4 (0.9%)	7 (1.6%)	13 (3.0%)	8 (1.8%)	11 (2.5%)	5 (1.1%)	40 (9.2%)	5 (1.1%)
**Females**	2,678	456 (17.0%)	314 (11.7%)	108 (4.0%)	52 (1.9%)	706 (26.4%)	99 (3.7%)	232 (8.7%)	24 (0.9%)	92 (3.4%)	62 (2.3%)	31 (1.2%)	21 (0.8%)	312 (11.7%)	41 (1.5%)	117 (4.4%)	11 (0.4%)
IC	1,683	277 (16.5%)	200 (11.9%)	50 (3.0%)	22 (1.3%)	439 (26.1%)	65 (3.9%)	122 (7.2%)	16 (1.0%)	68 (4.0%)	46 (2.7%)	24 (1.4%)	18 (1.1%)	237 (14.1%)	28 (1.7%)	64 (3.8%)	7 (0.4%)
MM	805	136 (16.9%)	85 (10.6%)	46 (5.7%)	18 (2.2%)	233 (28.9%)	30 (3.7%)	85 (10.6%)	7 (0.9%)	21 (2.6%)	12 (1.5%)	4 (0.5%)	3 (0.4%)	62 (7.7%)	11 (1.4%)	49 (6.1%)	3 (0.4%)
EX	190	43 (22.6%)	29 (15.3%)	12 (6.3%)	12 (6.3%)	34 (17.9%)	4 (2.1%)	25 (13.2%)	1 (0.5%)	3 (1.6%)	4 (2.1%)	3 (1.6%)	0 (0.0%)	13 (6.8%)	2 (1.1%)	4 (2.1%)	1 (0.5%)

Values in brackets represent frequencies (%) by row. IC, Individual contributors; MM, Middle managers; EX, Executives.

**Figure 1:**
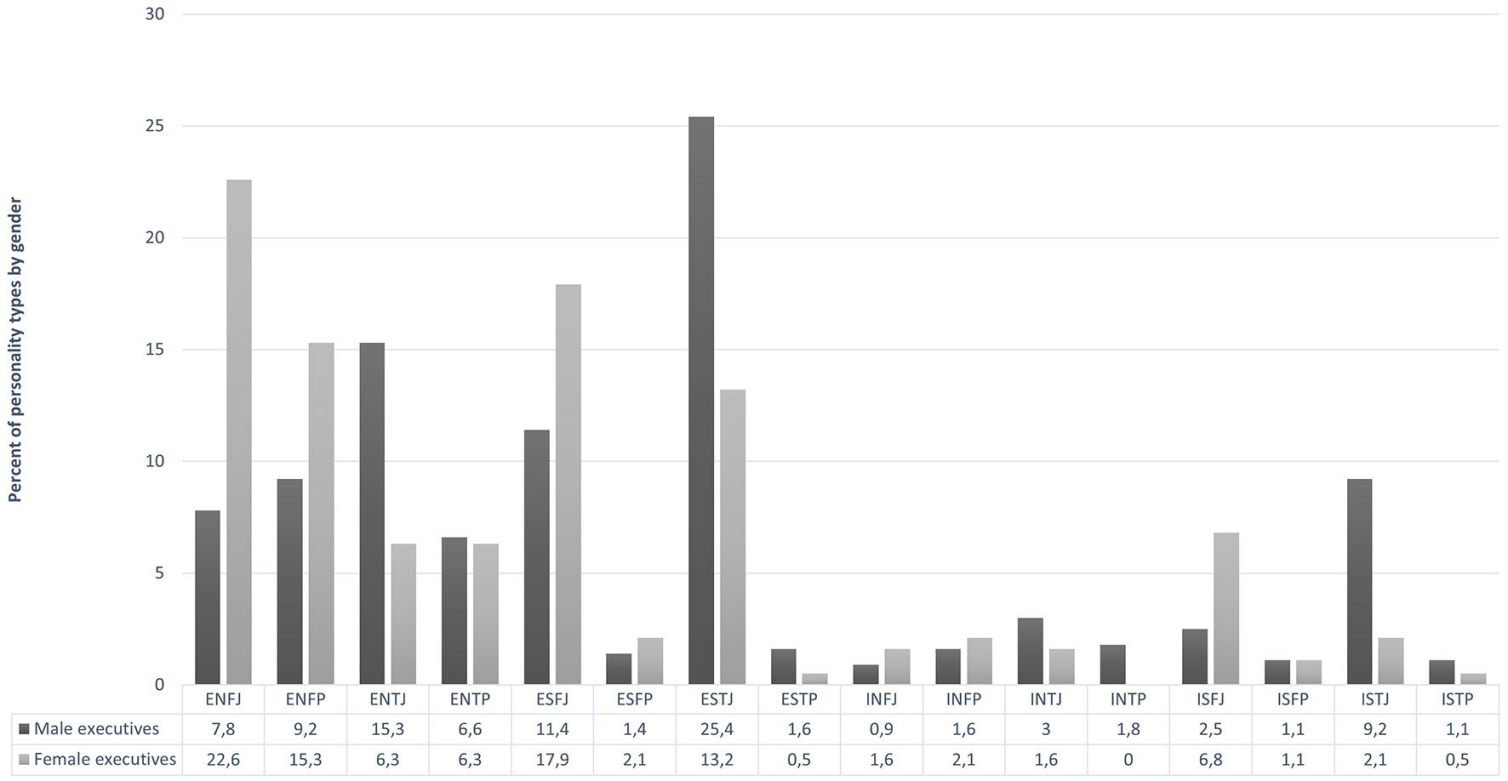
Frequency distribution of personality types of male and female executives. Numbers indicate percent of personality types by gender.

The chi-square statistics were significant for each hierarchical level and for the total sample as well: χ^2^ (15) = 486.944, *p* < 0.001 for individual contributors, χ^2^ (15) = 263.577, *p* < 0.001 for middle managers, χ^2^ (15) = 73.338, *p* < 0.001 for executives, and χ^2^ (15) = 878.208, *p* < 0.001 for the total sample. These results indicate gender differences in preferences regardless of hierarchical levels (see also the findings of the two-way ANOVAs), but also gender-related differences between successful males and females (i.e., executives). Figure 1 shows the frequencies of personality types of executives by gender. The most frequent personality types were ENFJ (22.6%), ESFJ (17.9%), ENFP (15.3%), and ESTJ (13.2%) among female executives, and ESTJ (25.4%), ENTJ (15.3%), ESFJ (11.4%), ENFP (9.2%), and ISTJ (9.2%) among male executives.

The distribution of personality types among male executives exhibits a greater degree of heterogeneity, resembling the type frequencies observed among middle managers and individual contributors, as delineated in Table 3. This contrasts with the pattern evident among women.

## 4. Discussion

Our study aimed to discern differences in corporate hierarchy levels among men and women, considering Jungian preferences. We identified significant disparities between employees across various hierarchical levels. Executives exhibited higher scores in Extraversion, Intuition, Thinking, Perceiving, and Calm compared to both middle managers and individual contributors. Contrary to our expectations, Judgment preferences were the highest among middle managers, and there were no differences between the scores of individual contributors and executives. These findings are broadly in line with prior research (Gardner and Martinko, 1996; Moutafi et al., 2007) except for Judgment (see Gardner and Martinko, 1996).

Another objective was to probe gender-related preference variances among leaders within the Hungarian context. Hungary's predominantly masculine culture and entrenched conservative gender role attitudes (e.g., Nagy and Vicsek, 2014; Scharle, 2015) create a challenging landscape for women striving for success in organizational realms. Our findings indicated that the proportion of female leaders is notably lower than that of male leaders. This outcome is in line with the literature showcasing the underrepresentation of women in leadership roles in Hungary (Nagy, 2012; Nagy et al., 2022).

Moreover, we found that females are more likely to succeed when exhibiting personality preferences (extraversion, intuition, feeling) that stereotypically align with the female leadership archetype (Saint-Michel, 2018). In contrast, men appear to have a more flexible trajectory toward higher hierarchy levels. Notably, female participants with an introverted preference were markedly absent from the executive group (with only 30 introverted female executives), while 94 men with this preference managed to attain executive positions.

Furthermore, Feeling and Thinking scores of female and male participants were also different at all hierarchical levels. Previous studies on management (for an overview see Gardner and Martinko, 1996) concluded that the Thinking preference was more common among women leaders compared to women in general. Although female executives indeed exhibited slightly higher Thinking and marginally lower Feeling scores than female middle managers and individual contributors, the overall gender-related pattern remained consistent. This finding underscores that women in Hungary are still expected to display higher levels of Feeling and lower levels of Thinking, even in executive roles.

While speculative, our results suggest that for men, leadership attainment is the norm. Men have a reasonable chance of success across a spectrum of personality preferences as they ascend the hierarchy. Conversely, for women, achieving leadership positions is an exception requiring specific preferences. They are more likely to possess either stereotypically feminine preferences (intuitive and feeling) or preferences resembling those commonly seen among male leaders (extraverted, sensing, thinking, and judging combination). Notably, women without these preferences face minimal prospects of reaching higher hierarchy levels.

Our findings have several theoretical and practical implications and could be of particular interest to policymakers.

The theoretical implication of our study is that the dilemmas of the literature on gender in management are very similar in the understudied Central and Eastern European context to the more frequently studied Western context. There is an ongoing debate about whether female leaders have a distinct female leadership style or not. This female leadership style is characterized as more inclusive, team-oriented, and transformative. However, there is a lack of cross-cultural studies on this topic (House et al., 2013). Our research demonstrates that females in the Hungarian context must conform to the stereotypical female leadership style, or fit into the traditional male leadership style, while their male counterparts can achieve success with a variety of personality preferences and types. The dilemmas are similar; however, the differences are more striking compared to the Western context. We did not only find gender-related differences in preferences among the members of the executive group (differently from earlier studies, e.g., Hansson and Andersen, 2007; Kummerow and Herk, 2011; Cohen et al., 2013; Gilal et al., 2016), but these differences were pervasive in our sample. In other words, females had strikingly different personality preferences compared to males regardless of their work status. These findings highlight the importance of culture and norms, and how these factors shape personality preferences.

The most important practical implication of the study relates to the different career trajectories and opportunities of men and women. There is an extensive discussion about the possible causes of women's underrepresentation in leadership (e.g., Hoyt, 2010). Many studies revealed that significant factors hindering women from advancing are stereotypes and implicit leadership theories that link masculinity to leadership and attribute a higher level of emotionality to women (Tharenou, 1999; Fischbach et al., 2015). The Thinking-Feeling preference patterns of this study highlight both: the generally stronger preference for Feeling among women compared to men and the increasing preference toward Thinking on higher hierarchy levels.

With the evolving approach toward women in leadership roles, there is a growing discourse on the potential advantages that female leaders could bring to organizational effectiveness, provided they are more widely represented in leadership positions (Powell et al., 2008). The relationship orientation requisite at managerial levels, increasingly emphasized in contemporary leadership style theories (Anzengruber et al., 2017), aligns with Feeling preferences according to Jungian theory (1971/1921). Hence, fostering a greater prevalence of this preference within leadership can potentially enhance overall leadership effectiveness.

Acknowledging the disparate opportunities available to Hungarian males and females in ascending to high-status positions, as well as recognizing the benefits of a diverse leadership landscape, we contend that addressing gender inequality within this context is of paramount practical importance. While Jungian preferences are generally deemed stable over time (Jung, 1971), recent evidence suggests that personality preferences can undergo change through interventions and major life events (Bleidorn et al., 2019). Consequently, it may be theoretically possible for females aspiring to leadership roles to mold their preferences to better align with leadership positions. Nonetheless, such an approach is neither feasible nor fair. We posit that the crux of the matter lies in institutional and societal structures rather than being a problem for the individual to solve. A strategy for the promotion of gender equality needs to be created with concrete, actionable steps and should put an emphasis on creating equal chances for females to achieve leadership positions as males.

Certain limitations warrant a mention. First, the nature of our data is descriptive, and it is based entirely on the self-descriptions of the participants. Moreover, explanatory and contingency variables were not included in the study. For instance, we did not collect data on perceived cultural norms and normative pressure, industry type, nature of tasks, or gender composition in the participants' teams. These factors are known to influence females' willingness to lead, and their effectiveness as a leader (e.g., Chen and Houser, 2019). A related limitation is that the data were collected over a relatively long period of time, during which many uncontrollable influences may have affected societal perceptions of female leaders. Unfortunately, for most subjects, we only know that they completed the GPOP test at some point between 2006 and 2018, but not precisely when. The date of completion was, therefore, not taken into account in the analyses. However, we agree with an anonymous reviewer's thoughtful observation, who pointed out that some of the observed differences could be simply due to the passage of time. Taken together, these limitations prevent causal inferences. Future studies should consider the impact of these explanatory and contingency variables.

Of particular interest is the career path of males and females. Our study speculatively suggests that males and females have different career paths in Hungary. For men, becoming a leader is the norm, while it seems that the female executive is the exception that proves the rule. However, longitudinal studies should investigate the changes in personality preferences during one's career path.

## Data availability statement

The raw data supporting the conclusions of this article will be made available by the authors, without undue reservation.

## Ethics statement

The studies involving humans were approved by United Ethical Review Committee for Research in Psychology (EPKEB), Hungary. The studies were conducted in accordance with the local legislation and institutional requirements. The participants provided their written informed consent to participate in this study.

## Author contributions

ES, AC, RB, and OK contributed to the conception and design of the study. The data collection was conducted by RB. ZS conducted the statistical analysis. ES, AC, and ZS drafted the manuscript. RB and OK contributed to finalizing the manuscript. RB provided language editing. All authors contributed to the article and approved the submitted version.
